# Strain Rate Behavior in Tension of Reinforcing Steels HPB235, HRB335, HRB400, and HRB500

**DOI:** 10.3390/ma9121013

**Published:** 2016-12-15

**Authors:** Feng Lin, Yu Dong, Xinxin Kuang, Le Lu

**Affiliations:** 1 Department of Structural Engineering, Tongji University, 1239 Siping Road, Shanghai 200092, China; dongyu_01@126.com (Y.D.); kuangxinxin2006@163.com (X.K.); 2 Key Laboratory of Advanced Civil Engineering Materials, Tongji University, 1239 Siping Road, Shanghai 200092, China; 3 Shanghai Dushi Green Engineering Co., Ltd., 2880 Shenkun Road, Shanghai 20110, China; Lule@dushigreen.com

**Keywords:** reinforcing steel bar, rebar, strain rate, impact, constitutive model

## Abstract

The strain rate effect of reinforcing steel bars is generally indispensable for modeling the dynamic responses of reinforced concrete structures in blast and impact events. A systematic experimental investigation was conducted on the strain rate behavior of reinforcing steel bar grades HPB235, HRB335, HRB400, and HRB500 which are widely used in the field of civil engineering in China. The dynamic testing was performed using a servo-hydraulic Instron VHS160/100-20 in a strain rate range from 2 to 75 s^−1^. Stress-strain curves at preset strain rates were obtained. The test data were then used to derive the parameters in a model based on the dynamic increase factors (DIFs) of strengths and the Johnson–Cook constitutive model. Results indicated that a significant strain rate effect was observed for the four rebar grades. The dynamic yield strengths increased from 13% to 41% and their ultimate strengths improved from 9% to 19% in the strain rate range during testing. The strain rate behavior of the four rebar grades could be appropriately predicted using the parameters in the model based on the DIFs of strengths and the Johnson–Cook model.

## 1. Introduction

Blast loading and impact action are occasionally considered for some structures in the field of civil engineering. Typical scenarios include a nuclear containment subjected to an aircraft crash, a control room in a chemical plant under blast loading, protective structures, and bridge piers impacted by ships. The involved facilities are usually constructed using reinforced concrete, due to its excellent blast resistant performance [1]. Consequently, high strain rates can occur in materials made of reinforcing steel bars (also called rebars) and concrete after being subjected to blasting and other severe dynamic loads. In case of such events, similar to concrete and other metallic materials, reinforcing steel bars exhibit a strain rate effect, which means their mechanical properties are different from those under static load conditions. Generally, the strain rate effect of rebars should be considered in the dynamic analyses of reinforced concrete structures.

Efforts have been made to investigate the strain rate behavior of various types of steel alloys and a few types of reinforcing steels in terms of strengths and deformation capacities [2,3,4,5,6,7,8,9,10]. Mainstone [2] summarized a quantity of test results for several types of steels before 1975 with strain rates $\dot{\varepsilon }$ ranging from 10^−2^ to 10^3^ s^−1^. He used dynamic increase factors (DIF) based on the ratio of dynamic strength to static strength with connections to strain rates to describe the strain rate effect. After that, investigations on the strain rate effect continued. Hu et al. recently demonstrated strain rate dependent behavior of AerMet 100 steel, which had excellent mechanical properties [4]. They used a Split-Hopkinson pressure bar which could generate strain rates in a range from 560 to 4200 s^−1^. In addition, the effects of strain rates on the tensile properties of a TRIP (transformation induced plasticity) -aided duplex stainless steel were studied by Choi et al. [5] using the Instron 4484 hydraulic testing machine with strain rates of 10^−3^, 5 × 10^−2^, and 10^−2^ s^−1^. On the other hand, for reinforcing steel bars that are widely used in concrete structures, relatively few studies were found in the literature. Early experimental studies were conducted by Brandes et al. [6] in 1986. They tested the reinforcing steel bars BSt 420/500 RU (hot rolled) and BSt 420/500 RK (cold draw) which were broadly used in Germany at that time. These test data were then adopted to build DIF expressions by Eibl [11]. Later, Malvar [12] presented a review of rebar properties with consideration of strain rates that varied from 10^−4^ to 10 s^−1^, and the yield strengths of the rebars were in the range of 290 to 710 MPa. He also proposed DIF formulations to describe the strain rate effect in terms of rebar strengths. More recently, the strain rate behavior of several particular types of reinforcing steel bars were investigated, including cold formed steel B500A ($\dot{\varepsilon }$ = 250, 500, and 1000 s^−1^) [13], quenched and self-tempered reinforcing steel B450C ($\dot{\varepsilon }$ = 250, 500, and 1000 s^−1^) [7], stainless steel AISI304 ($\dot{\varepsilon }$ = 10^−3^, 5, 30, 250, 500, 1000 s^−1^) [8], and low-alloy structural steel S355 ($\dot{\varepsilon }$ = 10^−3^, 5, 35, 300, 500, and 850 s^−1^) [9]. Based on these studies, the following observations could be made for steel alloys and rebars under high strain rates:

- An increase in yield strengths and ultimate strengths have been confirmed;
- Yield strength is more strain rate-sensitive than ultimate strength;
- Steels with relatively low strengths are susceptible to strain rates compared to those with relatively high strengths;
- No changes have been found in the elastic modulus;
- The magnitude of the strain rate effect is different for various types of steel materials;
- The variation tendency of plateau lengths (only for hot rolled rebars) and ultimate strains under high strain rates are inconsistent based on different test sources.

The reinforcing steel bars HPB235, HRB335, HRB400, and HRB500 are different rebar grades and have been widely used in reinforced concrete structures in China. Their strain rate behavior has not been systematically and experimentally investigated so far. These four types of rebars belong to hot rolled and low-carbon structural steels and possess good properties of strength, ductility, and processability. The rebars HPB235 and HRB335 are usually used as stirrups to resist shear forces, while rebars HRB400 and HRB500 commonly serve as longitudinal reinforcements to avoid bending failure. For each steel grade, the number (e.g., 235 in HPB235) denotes the yield strength used for design. As a matter of fact, the analyses of reinforced concrete structures under blasting and impact loads were short of full confidence due to the lack of the appropriate constitutive models of rebars. Evidently, the test results of other rebars in the literature were only of reference value for understanding the strain rate behavior of the rebars HPB235, HRB335, HRB400, and HRB500, because the dynamic behavior of steels is type-dependent. In this sense, to fill the current knowledge gap and enrich the database, the strain rate behaviors of rebars HPB235, HRB335, HRB400, and HRB500 were experimentally investigated in this study. The strain rates in testing ranged from 2 to 80 s^−1^, which are the strain rates that typically occur in blast [14,15] and impact events [10]. The parameters in DIF formulations and in the Johnson-Cook model were derived, and the results were compared with existing formulations. The primary objective of this study was to propose realistic material models of rebars for their application in dynamic analyses of reinforced concrete structures. Therefore, engineering stress and engineering strain were used throughout this paper.

## 2. Experimental Procedure

**Figure 1:**
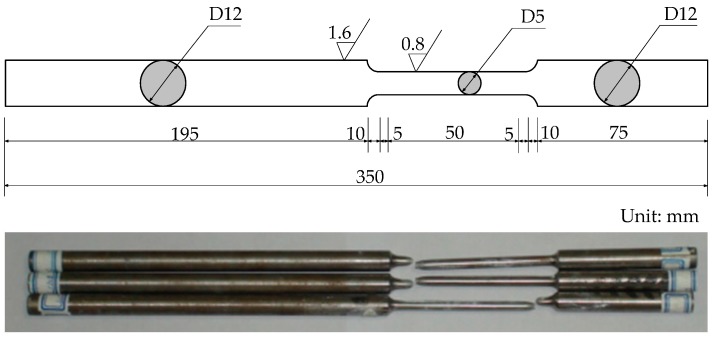
Specimen geometry.

Tensile testing was performed for rebar samples HPB235, HRB335, HRB400, and HRB500 at four strain rates using two test facilities at room temperature. Quasi-static testing was carried out using a universal electromechanical CSS-44500 testing machine (Changchun Research Institute for Mechanical Science Co., Ltd., Changchun, China) with a maximum load bearing capacity of 100 kN. Dynamic testing was conducted using a servo-hydraulic Instron VHS160/100-20 (Instron, Grove City, PA, USA) with a maximum load bearing capacity of 100 kN. This machine is particularly designed for elevated strain rate testing with preset tensile speeds. Round dumbbell-shape samples were fabricated with 50 mm length in the middle portion and 5 mm in diameter, as illustrated in Figure 1. These specimens were processed using rebar grades for HPB235, HRB335, HRB400, and HRB500 of 12, 14, 25, and 22 mm diameters, respectively, due to the limited load bearing capacity of the test machine. The strain rates ranged from 2 to 80 s^−1^ and were preset to three strain rate levels. For each preset strain rate level, three specimens were tested for the individual rebar grades to minimize the result variation. Table 1 presents the main chemical composition of the four rebar grades.

**Figure 2:**
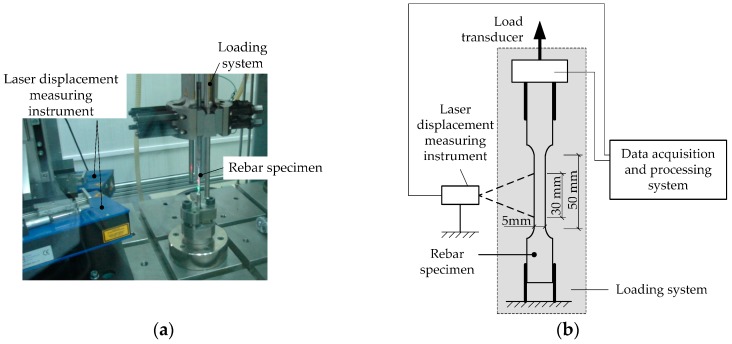
Test system for reinforcing steel bars under elevated strain rates: (**a**) Instron VHS160/100-20 and an installed specimen and (**b**) schematic diagram of testing.

**Figure 3:**
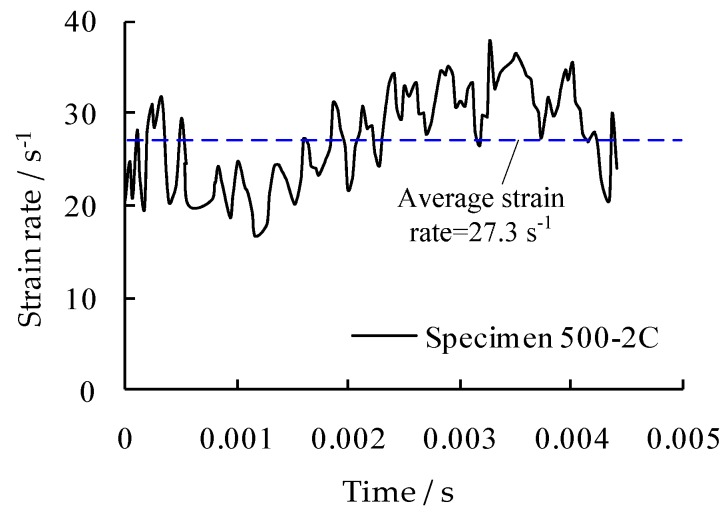
Strain rate vs. time curve for specimen 500-2C.

The quasi-static tests were conducted with a strain rate of less than 0.0003 s^−1^, in accordance with Chinese standards [16]. Figure 2 presents the test system for reinforcing steel bars under elevated strain rates. The specimen was clamped at both ends and tensioned under an approximate constant strain rate to failure. Figure 3 presents an example of the resulting strain rates varied within a small range during testing for specimen 500-2C. Particularly, a laser displacement measuring instrument was used to detect the length changes within a gauge length of 30 mm with a sufficient high frequency of 390 kHz. A load transducer in the test system recorded the tensile loads. The data were in a real-time historical form, and the output was from a data collection and processing system that could take in various forms including stress-strain curves.

A scanning electron microscope (SEM) FEI QUANTA650 (FEI Company, Hillsboro, OR, USA) was used to observe the microtopography of the fractured surfaces of the specimens. For each sample after rupture, a rebar portion, including the fractured surface, was cut from the sample and placed in absolute alcohol to prevent rust. The observation location was near the center of the fractured surface for each sample.

## 3. Test Results and Discussion

**Figure 4:**
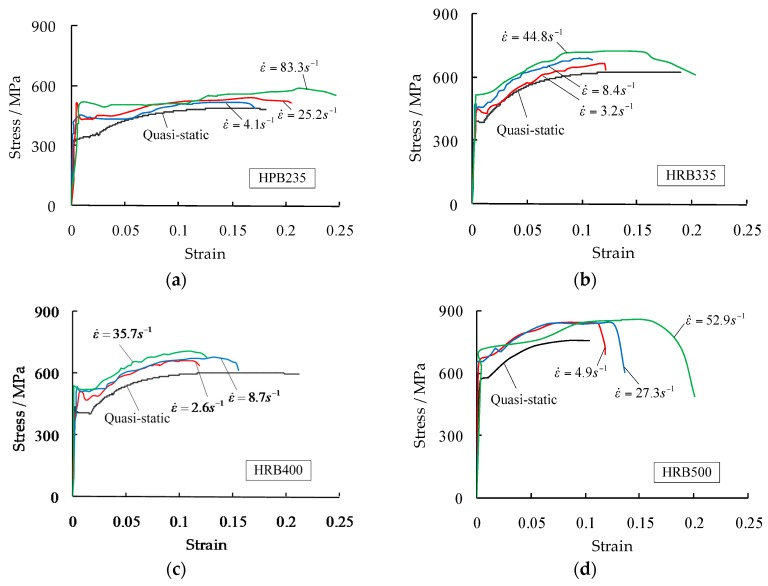
Typical stress-strain curves for the four rebar grades at different strain rates: (**a**) HPB235; (**b**) HRB335; (**c**) HRB400; and (**d**) HRB500.

Figure 4 presents the typical stress-strain curves for the four rebar grades at different strain rates. These were data filtered from the raw results after cutting off high-frequency noise. Table 2 summarizes the test results in terms of yield strength, ultimate tensile strength, ultimate tensile strain, and the corresponding DIFs. In each test group, the measured strain rates were slightly varied based on the checked calculations after individual testing. Average values in each group consisted of three tests, which are also presented. Results revealed that:

- The yield strengths and ultimate strengths increase with the increase in strain rates in the ranges from 13% to 41% and from 9% to 19%, respectively;
- The increased magnitudes of the yield strengths are greater than those of the ultimate strengths;
- The rebar grades with relatively low strengths (e.g., HPB235) are more susceptible to strain rates than the rebar grades with relatively high strengths (e.g., HRB500);
- Elastic moduli are independent of strain rates;
- No statistical tendency was found for the plateau lengths;
- No statistical tendency was found for the ultimate strains and all the rebars failed in a ductile manner.

**Figure 5:**
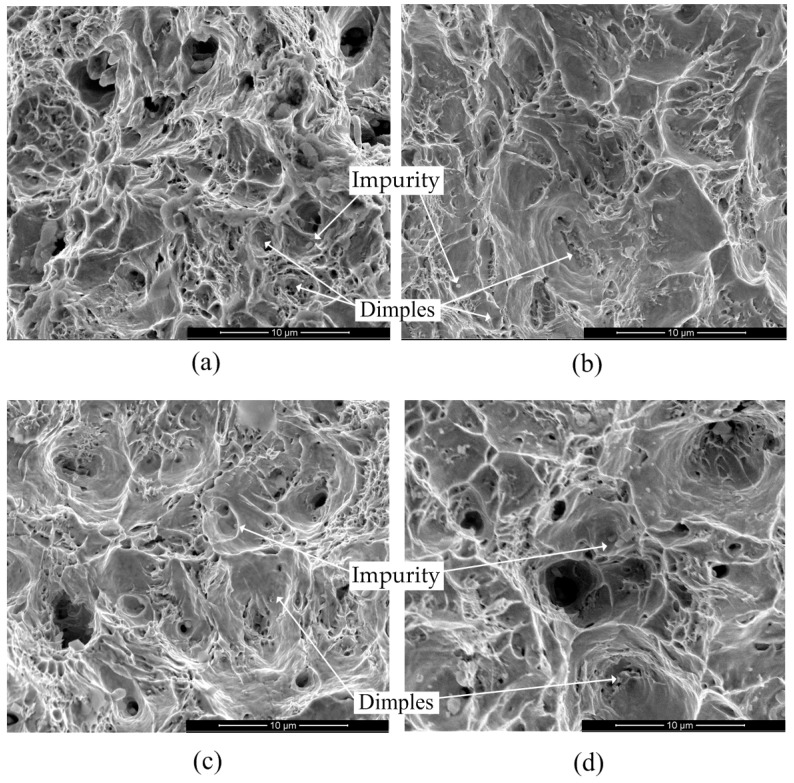
Fracture surface morphology of specimens of rebar grade HRB500 at different strain rates: (**a**) 500-0A ($\dot{\varepsilon }$ = 0.0003 s^−1^); (**b**) 500-1A ($\dot{\varepsilon }$ = 5.6 s^−1^); (**c**) 500-2A ($\dot{\varepsilon }$ = 23.8 s^−1^); and (**d**) 500-3A ($\dot{\varepsilon }$ = 52.9 s^−1^).

These results were qualitatively consistent with the previous understanding relating to other rebars. These data built the basis for calibrating the parameters in empirical models with consideration of the strain rate effect. As an example, Figure 5 illustrates the fractured surface morphology of rebar grade HRB500 at different strain rates. The fracture features a typical toughness fracture with microporous accumulation, which means the materials failed in a ductile manner and is consistent with the observations in testing. Dimples were found that had sharp boundaries and various sizes and heights. Shear lines were also observed in the internal wall surface of some big dimples. Impurity of *Fe* was detected in a small number of dimples using energy spectrum analysis.

## 4. Constitutive Models

Two commonly used constitutive models were adopted to describe the strain rate behavior of the rebars. One was the model based on the DIFs of strengths [11,12] in relation to strain rates. By using DIF formulations, the dynamic material model of the rebars in forms of a function, e.g., bilinearity or trilinearity, could be forward built. The other was the well-known Johnson-Cook (JC) model [17], which was adopted in commercial finite element method (FEM) analysis codes. Both constitutive models have applications in the numerical simulation of reinforced concrete structures subjected to blast loads [15,18]. In addition, the average values of the yield and ultimate strengths in each test group in Table 2 were used to derive the parameters in the constitutive models.

### 4.1. DIF Formulations

The DIFs for the yield strengths, *f*_y,d_/*f*_y,st_, and for the ultimate strengths, *f*_u,d_/*f*_u,st_, of the rebars can be expressed as:

(1) $$\frac{f_{y,d}}{f_{y,\text{st}}}=1+\frac{D_{1}}{f_{y,\text{st}}}\;\text{ln}\;\frac{\dot{\varepsilon }}{{\dot{\varepsilon }}_{0}}$$

(2) $$\frac{f_{u,d}}{f_{u,\text{st}}}=1+\frac{D_{2}}{f_{u,\text{st}}}\;\text{ln}\;\frac{\dot{\varepsilon }}{{\dot{\varepsilon }}_{0}}$$

where *f*_y,d_ and *f*_y,st_ represent the yield strength of individual rebars under dynamic and static loading, respectively; *f*_u,d_ and *f*_u,st_ are the ultimate strengths of individual rebars under dynamic and static loading, respectively; $\dot{\varepsilon }$ denotes the strain rate, and ${\dot{\varepsilon }}_{0}$ = 0.0003 s^−1^ is the strain rate under quasi-static loading. *D*_1_ and *D*_2_ are two parameters which are determined using linear regression analysis and result in *D*_1_ = 10.05, 8.73, 8.72, and 9.72 MPa, and *D*_2_ = 6.38, 6.54, 6.54, and 7.78 MPa for reinforcing steel bars HPB235, HRB335, HRB400, and HRB500, respectively.

### 4.2. Johnson–Cook Model

The Johnson–Cook model is based on three independent phenomena, i.e., isotropic hardening, strain-rate hardening, and thermal softening. Only the isotropic hardening and strain-rate hardening were considered in this study. Thermal softening was not studied in the present tests due to the difficulty to monitor the temperature variation during loading. Similar consideration was also adopted in references [4,7,8,13]. As a result, the stress, *σ*, can be expressed as:

(3) $$\sigma =[A+B{\left(\varepsilon_{p}\right)}^{n}][1+C\text{ln}\;\frac{{\dot{\varepsilon }}_{p}}{{\dot{\varepsilon }}_{0}}]$$

where *ε*_p_ is the equivalent plastic strain; ${\dot{\varepsilon }}_{p}$ is the strain rate under consideration; ${\dot{\varepsilon }}_{0}$ represents the reference strain rate of 1 s^−1^; and parameters *A*, *B*, *n*, and *C* are the material constants to be determined using the test data. The parameters *B* and *n* are related to the strain-hardening, while *C* represents the strain-rate sensitivity. Table 3 presents the obtained parameters *A*, *B*, *n*, and *C*.

### 4.3. Comparison with Existing Formulations

Table 4 presents the comparison among DIFs of yield and ultimate strengths at average strain rates in tests obtained from different sources: (i) the testing; (ii) Equations (1) and (2); (iii) Equation (3); (iv) formulations in [11]; and (v) Malvar’s formulations in [12]. The formulations in [11] for hot-rolled reinforcing steel with yield strength for design of 420 MPa are expressed as:

(4) $$\frac{f_{y,d}}{f_{y,\text{st}}}=1+\frac{6.0}{f_{y,\text{st}}}\;\text{ln}\;\frac{\dot{\varepsilon }}{5\times 10^{-5}}$$

(5) $$\frac{f_{u,d}}{f_{u,\text{st}}}=1+\frac{7.0}{f_{u,\text{st}}}\;\text{ln}\;\frac{\dot{\varepsilon }}{5\times 10^{-5}}$$

The formulations proposed by Malvar are [12]:

(6) $$\frac{f_{y,d}}{f_{y,\text{st}}}={\left(\frac{\dot{\varepsilon }}{10^{-4}}\right)}^{0.074-0.040\frac{f_{y,st}}{60}}$$

(7) $$\frac{f_{y,d}}{f_{y,\text{st}}}={\left(\frac{\dot{\varepsilon }}{10^{-4}}\right)}^{0.019-0.009\frac{f_{y,st}}{60}}$$

where *f*_y,st_ is required in ksi and the expressions are applicable for strain rates up to 10 s^−1^ in Equations (6) and (7). In the comparisons, the formulations in [11] were used only for rebar grade HRB400 with yield strength for design of 400 MPa. Malvar’s formulations were applied with a limitation of strain rates no more than 10 s^−1^.

**Figure 6:**
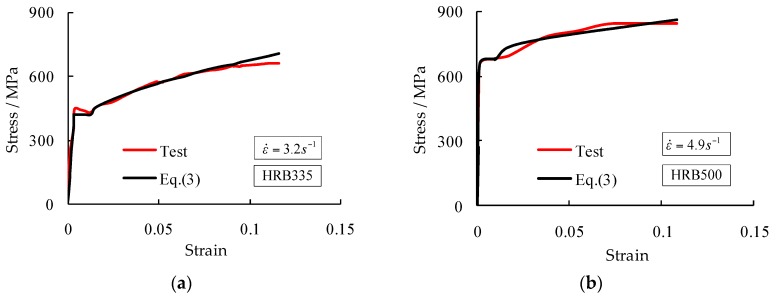
Typical stress-strain curves obtained from testing and Equation (3) for rebar grade HRB335 at a strain rate of 3.2 s^−1^ (**a**) and for rebar grade HRB500 at a strain rate of 4.9 s^−1^ (**b**).

Results found that Equations (1)–(3) well predicted the test data with a maximum error of about 21%. Formulations in [11] could be used to predict the DIFs of rebar grade HRB400 with an error no more than 7%. Malvar’s formulation for the DIF of ultimate strength appropriately matched the test results, whilst the formulation for the DIF of yield strength overestimated the strain rate effect observed in testing. As examples, Figure 6 presents the typical stress-strain curves obtained from testing and Equation (3) for rebar grade HRB335 at a strain rate of 3.2 s^−1^ and for rebar grade HRB500 at a strain rate of 4.9 s^−1^. Good agreement was achieved.

Generally, Equations (1)–(3) can be used to describe the strain rate effect of rebar grades HPB235, HRB335, HRB400, and HRB500 with strain rates in testing of $2\leq \dot{\varepsilon }\leq 75$ s^−1^. However, these equations are also suggested to apply for those with $\dot{\varepsilon }\leq 2$ s^−1^ based on check calculations and the details are not presented for brevity.

## 5. Conclusions

Strain rate tests were conducted to investigate the strain rate effect of reinforcing steel bars HPB235, HRB335, HRB400, and HRB500. The stress-strain relationships at different strain rates in a range of $2\leq \dot{\varepsilon }\leq 75$ s^−1^ were obtained. The test data were then used to calibrate the parameters in a model based on the DIFs of strengths, and the JC model, both of which are frequently used in simulations of the dynamic performance of reinforced concrete structures. The following conclusions were drawn:

(1) Significant strain rate effect was observed for the four rebar grades. Their yield strengths increased from 13% to 41% and their ultimate strengths improved from 9% to 19% in the strain rate range in the testing.
(2) The strain rate behavior of the four rebar grades could be appropriately predicted using the parameters in the model based on the DIFs of strengths and the JC model for strain rates less than 75 s^−1^.

## Figures and Tables

**Table 1:** Chemical composition (wt.%) of the four rebar grades.

Rebar Grade	C	S	Mn	Si	P	Cr	V	Mo	Cu	Ni
HPB235	0.15	0.025	0.53	0.16	0.029	-	-	-	-	-
HRB335	0.20	0.026	1.41	0.50	0.028	0.090	<0.02	<0.02	0.088	0.022
HRB400	0.22	0.027	1.38	0.52	0.023	0.031	<0.02	<0.02	<0.02	<0.02
HRB500	0.25	0.28	1.43	0.03	0.02	0.07	<0.01	0.10	0.02	0.02

**Table 2:** Test results.

Rebar Grade	Group	Specimen No.	$\dot{\varepsilon }$ (1/s)	*f*_y_ (Mpa)	DIF of *f*_y_	*f*_u_ (Mpa)	DIF of *f*_u_	Ultimate Strain ^#^
HPB235	235-0	235-0A	≤0.0003	329.0	-	484.0	-	0.141
		235-0B	≤0.0003	326.0	-	479.0	-	0.161
		235-0C	≤0.0003	332.0 (329.0) *	-	490.0 (484.0)	-	0.156 (0.153)
	235-1	235-1A	4.1	443.6	1.35	519.1	1.07	0.128
		235-1B	7.1	401.7	1.22	541.8	1.12	0.257
		235-1C	8.2 (6.5) *	417.1 (420.8)	1.27 (1.28)	556.0 (539.0)	1.15 (1.11)	0.205 (0.197)
	235-2	235-2A	22.4	429.7	1.31	565.0	1.17	0.257
		235-2B	15.4	432.6	1.32	540.8	1.12	0.167
		235-2C	25.2 (21.0)	445.7 (436.0)	1.36 (1.33)	542.5 (549.4)	1.12 (1.14)	0.179 (0.201)
	235-3	235-3A	68.4	443.9	1.35	564.1	1.17	0.235
		235-3B	73.0	455.7	1.39	572.1	1.18	0.165
		235-3C	83.3 (74.9)	495.1 (464.9)	1.51 (1.41)	588.6 (575.0)	1.22 (1.19)	0.213 (0.204)
HRB335	335-0	335-0A	≤0.0003	387.0	-	626.0	-	0.154
		335-0B	≤0.0003	383.0	-	631.0	-	0.153
		335-0C	≤0.0003	390.0 (386.7)	-	640.0 (632.3)	-	0.152 (0.153)
	335-1	335-1A	3.2	425.7	1.10	701.3	1.11	0.161
		335-1B	2.3	460.5	1.19	696.8	1.10	0.102
		335-1C	3.2 (2.9)	428.8 (438.3)	1.11 (1.13)	662.0 (686.7)	1.05 (1.09)	0.116 (0.126)
	335-2	335-2A	7.6	454.6	1.18	680.0	1.08	0.085
		335-2B	8.4	460.4	1.19	690.8	1.09	0.097
		335-2C	9.1 (8.4)	469.4 (461.5)	1.21 (1.19)	704.2 (691.7)	1.11 (1.09)	0.120 (0.101)
	335-3	335-3A	44.8	518.6	1.34	723.6	1.14	0.12
		335-3B	39.0	534.6	1.38	699.5	1.11	0.118
		335-3C	30.6 (38.1)	518.8 (524.0)	1.34 (1.35)	737.1 (720.1)	1.17 (1.14)	0.092 (0.110)
HRB400	400-0	400-0A	≤0.0003	403.0	-	604.0	-	0.156
		400-0B	≤0.0003	406.0	-	603.0	-	0.158
		400-0C	≤0.0003	402.0 (404.0)	-	602.0 (603.0)	-	0.153 (0.156)
	400-1	400-1A	2.6	473.0	1.17	662.9	1.10	0.107
		400-1B	3.0	485.3	1.20	653.4	1.08	0.116
		400-1C	3.5 (3.0)	492.2 (483.5)	1.22 (1.20)	635.9 (650.7)	1.06 (1.08)	0.130 (0.118)
	400-2	400-2A	9.8	500.0	1.24	654.5	1.09	0.132
		400-2B	9.5	498.1	1.23	654.1	1.09	0.126
		400-2C	8.7 (9.3)	512.0 (503.3)	1.27 (1.25)	679.0 (662.5)	1.13 (1.10)	0.132 (0.130)
	400-3	400-3A	49.9	520.0	1.29	689.6	1.14	0.118
		400-3B	35.7	526.7	1.30	707.3	1.17	0.109
		400-3C	35.8 (40.5)	517.0 (521.2)	1.28 (1.29)	694.4 (697.1)	1.15 (1.16)	0.117 (0.115)
HRB500	500-0	500-0A	≤0.0003	594.7	-	779.6	-	0.096
		500-0B	≤0.0003	572.7	-	752.1	-	0.097
		500-0C	≤0.0003	579.9 (582.4)	-	760.3 (764.0)	-	0.093 (0.095)
	500-1	500-1A	5.6	642.1	1.10	837.3	1.10	0.094
		500-1B	4.9	676.2	1.16	839.0	1.10	0.102
		500-1C	5.2 (5.2)	656.7 (658.3)	1.13 (1.13)	841.3 (839.2)	1.10 (1.10)	0.099 (0.098)
	500-2	500-2A	23.8	698.7	1.20	865.9	1.13	0.113
		500-2B	28.7	678.1	1.16	862.9	1.13	0.108
		500-2C	27.3 (26.6)	707.0 (694.6)	1.21 (1.19)	843.4 (857.4)	1.10 (1.12)	0.114 (0.112)
	500-3	500-3A	52.9	712.6	1.22	856.9	1.12	0.134
		500-3B	59.0	718.3	1.23	859.2	1.12	0.145
		500-3C	50.7 (54.2)	726.6 (719.2)	1.25 (1.23)	888.1 (868.1)	1.16 (1.14)	0.123 (0.134)

Note: * The numbers in parentheses are average values for each test group consisting of three specimens identified as A, B, and C. ^#^ Ultimate strain denotes the strain at maximum stress.

**Table 3:** Parameter values in the Johnson–Cook model.

Rebar Grade	*A* (MPa)	*B* (MPa)	*n*	*C*
HPB235	377.7	160.8	0.3731	0.093
HRB335	401.1	1167.7	0.6761	0.087
HRB400	468.9	1201.8	0.7094	0.034
HRB500	629.6	666.2	0.5976	0.030

**Table 4:** Comparison among DIFs of yield and ultimate strengths obtained from different sources.

Rebar Grade	Average Strain Rate (s^−1^)	DIF of Strength	DIF				
			Testing	Equations (1) and (2)	Equation (3)	Formulations in [11]	Malvar’s Formulations [12]
HPB235	6.5	DIF of *f*_y_	1.28	1.30	1.35	-	1.59
		DIF of *f*_u_	1.11	1.13	1.13	-	1.14
	21.0	DIF of *f*_y_	1.33	1.34	1.47	-	-
		DIF of *f*_u_	1.14	1.15	1.23	-	-
	74.9	DIF of *f*_y_	1.41	1.38	1.61	-	-
		DIF of *f*_u_	1.19	1.16	1.35	-	-
HRB335	2.9	DIF of *f*_y_	1.13	1.21	1.13	-	1.45
		DIF of *f*_u_	1.09	1.09	1.19	-	1.11
	8.4	DIF of *f*_y_	1.19	1.23	1.23	-	1.51
		DIF of *f*_u_	1.09	1.11	1.21	-	1.13
	38.1	DIF of *f*_y_	1.36	1.27	1.37	-	-
		DIF of *f*_u_	1.14	1.12	1.38	-	-
HRB400	3.0	DIF of *f*_y_	1.20	1.20	1.20	1.16	1.43
		DIF of *f*_u_	1.08	1.10	1.26	1.13	1.12
	9.3	DIF of *f*_y_	1.25	1.22	1.25	1.18	1.49
		DIF of *f*_u_	1.10	1.11	1.34	1.14	1.12
	40.5	DIF of *f*_y_	1.29	1.25	1.31	1.20	-
		DIF of *f*_u_	1.16	1.13	1.36	1.16	-
HRB500	5.2	DIF of *f*_y_	1.13	1.16	1.13	-	1.21
		DIF of *f*_u_	1.10	1.10	1.09	-	1.07
	26.6	DIF of *f*_y_	1.19	1.19	1.19	-	-
		DIF of *f*_u_	1.12	1.12	1.16	-	-
	54.2	DIF of *f*_y_	1.23	1.20	1.21	-	-
		DIF of *f*_u_	1.14	1.12	1.22	-	-
